# Comparisons of Prediction Models of Myofascial Pain Control after Dry Needling: A Prospective Study

**DOI:** 10.1155/2013/478202

**Published:** 2013-06-18

**Authors:** Yuan-Ting Huang, Choo-Aun Neoh, Shun-Yuan Lin, Hon-Yi Shi

**Affiliations:** ^1^Nursing Department, Kaohsiung Armed Forces General Hospital, Kaohsiung 80201, Taiwan; ^2^Section of Anesthesiology, Pingtung Christian Hospital, Pingtung 90059, Taiwan; ^3^Department of Healthcare Administration and Medical Informatics, Kaohsiung Medical University, No. 100, Shih-Chuan 1st Road, Kaohsiung 80708, Taiwan

## Abstract

*Background*. This study purposed to validate the use of artificial neural network (ANN) models for predicting myofascial pain control after dry needling and to compare the predictive capability of ANNs with that of support vector machine (SVM) and multiple linear regression (MLR). *Methods*. Totally 400 patients who have received dry needling treatments completed the Brief Pain Inventory (BPI) at baseline and at 1 year postoperatively. *Results*. Compared to the MLR and SVM models, the ANN model generally had smaller mean square error (MSE) and mean absolute percentage error (MAPE) values in the training dataset and testing dataset. Most ANN models had MAPE values ranging from 3.4% to 4.6% and most had high prediction accuracy. The global sensitivity analysis also showed that pretreatment BPI score was the best parameter for predicting pain after dry needling. *Conclusion*. Compared with the MLR and SVM models, the ANN model in this study was more accurate in predicting patient-reported BPI scores and had higher overall performance indices. Further studies of this model may consider the effect of a more detailed database that includes complications and clinical examination findings as well as more detailed outcome data.

## 1. Introduction

Myofascial pain syndrome (MPS), a common cause of musculoskeletal pain presenting in primary care, results from myofascial trigger point activity [1, 2]. Dry needling is a treatment modality that is minimally invasive, cheap, and easy to learn with appropriate training and carries a low risk. Its effectiveness has been confirmed in numerous studies and comprehensive systematic reviews [3–5]. Accurately predicting myofascial pain control, a standard outcome measure after dry needling, is important when selecting treatment modality and when allocating scarce medical resources [1, 2]. 

Regression analysis, one of the most widely used multivariate analysis methods, assumes linear relationships between independent and dependent variables. However, studies show that changes in biomedical variables are often nonlinear [6–10]. The major classifier methods use support vector machines (SVMs) to solve classification problems by constructing hyperplanes in a multidimensional space that separates cases of different class labels. However, SVMs have also been proven effective for solving regression problems because they can handle multiple continuous variables [6–10]. Artificial neural networks (ANNs) are complex and flexible nonlinear systems with properties not found in other modeling systems. These properties include robust performance in dealing with noisy or incomplete input patterns, high fault tolerance, and the capability to generalize from the input data [6–10]. The computational power of an ANN is derived from the distributed nature of its connections. The ANN model is a well-established data mining algorithm that is widely used in various fields, from engineering to biomedical science [6–10]. 

The multilayer perceptron (MLP) is the most frequently used ANN due to its ability to model nonlinear systems and establish nonlinear decision boundaries in classification problems such as optical character recognition, data mining, and image processing/recognition [11, 12]. Our chosen model in the present study was a multilayer perceptron network, and we focus on the MLP type of ANN, the most common type. 

Despite their contribution to the growing understanding for predicting myofascial pain control after dry needling, previous studies of dry needling outcome have had major shortcomings [13–15]. Few studies of dry needling outcome have used longitudinal data for more than one year. Moreover, no studies have considered group differences in factors other than outcome such as age and nonsurgical treatment. Additionally, almost all published articles agree that the essential issue of the internal validity (reproducibility) of the ANN, the SVM, and multiple linear regression (MLR) models has not been adequately addressed. 

Therefore, the primary aim of this study was to validate the use of ANN models in predicting patient-reported quality of life (QOL) after dry needling, and the secondary aim was to compare the predictive capability of ANNs with that of SVM and MLR models. 

## 2. Materials and Methods

### 2.1. Ethics Statement and Study Population

**Figure 1:**
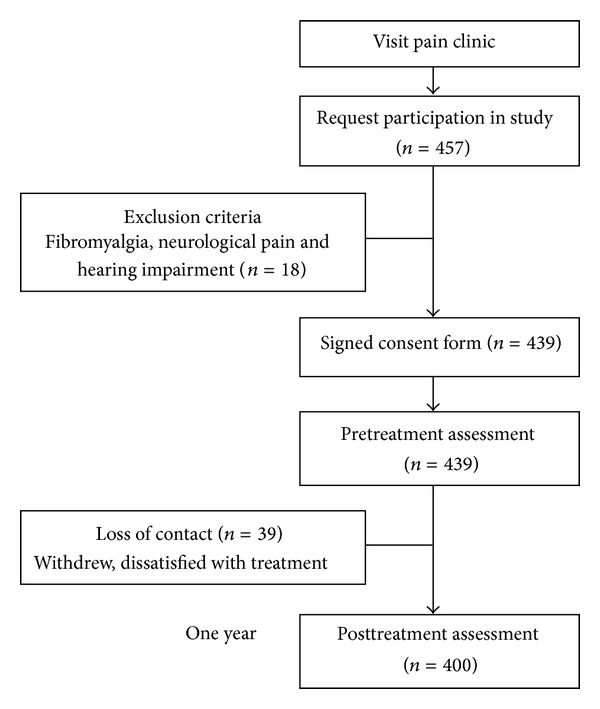
Progression of participants through the trial, including those who met exclusion criteria, those who withdrew, and those who were lost to followup.

The subjects included all MPS patients who had been referred for evaluation and treatment to the Pingtung Christian Hospital (PTCH) pain clinic from February to October, 2008. Inclusion criteria were chronic musculoskeletal pain for three months or longer due to nonspecific muscle pain, physical examination revealing tender spot in a palpable taut band, ability of a patient to distinguish between varying intensity of pain, referred pain pattern and local twitch response, Chinese speaking, and age at least 18 years. Exclusion criteria were fibromyalgia syndrome, neurological pain, infection, drug or alcohol abuse, rheumatologic disease, pregnancy, and any other disease that might interfere with participation (*n* = 18). The research protocol was reviewed and approved by the institutional review boards of PTCH. Of the 439 eligible subjects who gave written consent and were enrolled in the study at baseline, thirty-nine patients were excluded due to loss of contact. All 400 of myofascial pain control after dry needling subjects completed the pretreatment and 1-year posttreatment assessments (Figure 1).

### 2.2. Interventions

All needling protocols were performed by a single specialist. Taut bands with trigger points were isolated by palpation to ensure reproducibility of symptoms. Therapeutic needling was then performed with sterile 32G-diameter, 80 mm acupuncture needles. A needle plunger was first used to pierce the skin and muscle with the acupuncture needle. After the needle penetrated the skin, the plunger was removed, and the needle was inserted further into the taut band to elicit a twitch response. Appropriate placement of the needle was confirmed by reproduction of recognizable pain or by observation of local twitch response. The needle was then partially withdrawn and repeatedly inserted into the muscle until no further twitches were observed. After inactivating trigger points and reducing referred pain, the specialist then passively stretched the involved muscle toward its normal length. The patients then performed the muscle-stretch exercise technique developed by Travell and Simons [16]. All subjects received eight needling protocols administered over an 8-week period and no other treatment was given in the next months after 8-week dry needling. 

### 2.3. Instruments and Measurements

 After the dry needling protocol, each subject completed a questionnaire regarding demographic information, individual lifestyle, and pretreatment pain function. The questions about individual lifestyle assessed factors such as smoking, drinking, sleep deprivation, and nutritional inadequacies. The questionnaire assessed whether the subjects had smoked 100 or more cigarettes in their lifetimes, whether they currently smoked cigarettes every day or some days, whether they consumed 10–45 g of alcohol per day, whether they subjectively needed sleep 1 h > actual sleep time, and whether they had ever been diagnosed with a vitamin or iron deficiency [2, 17]. 

The Taiwan version of the Brief Pain Inventory (BPI-T) was used [2, 18]. The BPI-T developed from the original BPI measures intensity of pain (sensory dimension) and interference of pain in daily life (reactive dimension) on a simple numeric scale from 0 to 10. Pain intensity was assessed by a four-item self-reported inventory requiring patients to rate their pain at the time of completing the questionnaire (present pain) and also when it was “worst,” “least,” and “average” within the previous week. Due to no significant improvement in BPI least pain score between baseline and 1 year postoperatively, the present study finally did not predict least pain in multiple linear regression models after dry needling. Pain severity was measured on a scale from 0 (no pain) to 10 (extreme pain). A similar seven-item self-reported inventory was used to measure interference of pain with daily life, including general activity, mood, walking ability, normal work, relationships with others, sleep, and enjoyment of life. The anchor points for each of the interference scale items were “0” (“no interference”) and “10” (“extreme interference”). In addition to reporting present pain intensity, patients were instructed to indicate any changes in the type of pain and any use of nonpharmacological pain treatment. The coefficient alpha regarding internal reliability was 0.84 for the severity scale and 0.88 for the interference scale. 

All data collection was performed by the trained research assistant. Baseline data collection was as follows: pain questionnaire and BPI-T (both at pain clinic); follow-up BPI-T by telephone interview one year later. 

### 2.4. System Model Development

The factors used in the MLR model to predict 1-year pain function of dry needling patients included patient characteristics. The MLR model can be formulated as the following linear equation:

(1) $$\begin{array}{c} \hat{Y}=\beta_{0}+\beta_{i}X_{i}+\varepsilon_{i},\;i=1,2,\ldots ,m, \end{array}$$

where $\hat{Y}$ is the actual output value, *β*_0_ is the intercept, *β*_*i*_ is the model coefficient parameter, *X*_*i*_ is the independent or input variable, *ε*_*i*_ is the random error, and *m* is the number of variables. 

The SVM model employs nonlinear mapping to transform the original training data into higher-dimensional data and searches for the linear optima that define a hyperplane within the new dimension [8]. With appropriate nonlinear mapping to a sufficiently high dimension, a decision boundary can separate data into two classes [8]. In the SVM model, this decision boundary is defined by support vectors and margins. 

The ANN model used in this study was a standard feedforward, backpropagation neural network with three layers: an input layer, a hidden layer, and an output layer. The MLP network is an emerging tool for designing special classes of layered feedforward networks [19]. Its input layer consists of source nodes, and its output layer consists of neurons; these layers connect the network to the outside world. In addition to these two layers, the MLP usually has one or more layers of neurons referred to as hidden neurons because they are not directly accessible. The hidden neurons extract important features contained in the input data. 

### 2.5. Statistical Analysis

The dataset was divided randomly into two sets, one set of 320 cases (80% of the overall dataset) for training the model and another set of eighty cases for testing the model. The model was built using the training set. Demographic and clinical characteristics were the independent variables, and the pain function was the dependent variable. The SVM, MLR, and ANN models were then tested using the eighty cases in the testing dataset. 

The model fit and prediction accuracy of the system models were measured in terms of mean square error (MSE) and mean absolute percentage error (MAPE), respectively. The MSE, which is computed between the desired and predicted values and then averaged across all data, is used as an indicator of goodness of fit. The MAPE indicates the average deviation from the desired value and is usually expressed as a percentage [9, 19]. The prediction accuracy of a model is considered excellent if its MAPE value is lower than 10%. Values between 10% and 20%, between 20% and 50%, and higher than 50% are considered indicators of high, average, and low prediction accuracy, respectively [9, 19]. The formulas for calculating MSE and MAPE are

(2) $$\begin{array}{c} \text{MSE}=\frac{1}{n}\sum_{i=1}^{n}{\left(Y_{i}-{\hat{Y}}_{i}\right)}^{2},\\ \text{MAPE}=\frac{1}{n}\sum_{i=1}^{n}\frac{\left|Y_{i}-{\hat{Y}}_{i}\right|}{Y_{i}}\times 100\%, \end{array}$$

where *n* is the number of observations, *Y*_*i*_ is the desired (target) value of the *i*th observation, and ${\hat{Y}}_{i}$ is the actual output value of the *i*th observation.

The change rates are also given. The optimal number of neurons in the hidden layer and the activation functions are iteratively determined by comparing the MSE index of the output error among several neural networks. The network training process continues as long as training and test errors decrease. That is, training stops when the training error rate and test error rate no longer change or when they begin increasing. The prediction accuracy of the model is then judged by computing the MAPE value. The change rate is also used to compare model performance between the training and test sets. This criterion is used to calculate the difference in MSE index between the test and the training sets so that the better model can be identified. Absolute value was defined as [(the MSE value from test set − the MSE value from training set)/(the MSE value from training set)] × 100%. The lower the change rate and the lower the MSE value are, the better the model performs. 

The unit of analysis in this study was the individual MPS patients after dry needling. The data analysis was performed in several stages. Firstly, continuous variables were tested for statistical significance by one-way analysis of variance (ANOVA), and categorical variables were tested by Fisher's exact analysis. Univariate analyses were applied to identify significant predictors (*P* < 0.05). Secondly, STATISTICA 10.0 (StatSoft, Tulsa, OK, USA) software was used to construct the MLP network model, the SVM model, and the MLR model of the relationship between the identified predictors and pain function. Finally, to simplify the training process, key variables were introduced, and unnecessary variables were excluded. A global sensitivity analysis was also performed to assess the relative significance of input parameters in the system model and to rank the variables in order of importance. The global sensitivity of the input variables against the output variable was expressed as the ratio of the network error (variable sensitivity ratios, VSR) with a given input omitted to the network error with the input included. A ratio of 1 or lower indicates that the variable degrades network performance and should be removed.

## 3. Results

Table 1 shows the patient's characteristics in this study. The mean age of the study population was 48.57 years (standard deviation, SD = 12.63 years). The average pain duration was 42.53 months (SD = 40.21 months), and 71.0% of the patients were female.  Table 2 shows the coefficients for worst pain, average pain, present pain, and aggregated pain interference obtained by the training set in the MLR model. The selected variables included in the MLR models were age, pain duration, gender, marital status, sleep deprivation, nutritional deficiency, and pretreatment BPI score. All the selected variables were statistically significant (*P* < 0.05).

Table 3 shows the three-layer networks and number of support vectors of worst pain, average pain, present pain, and aggregated pain interference in ANN and SVM models. The ANN-based approaches provided the 3-layer networks and the relative weights of neurons used for predicting BPI score. The activation functions of logistic sigmoid and hyperbolic tangent were used in each neuron of the hidden layer and output layer, respectively.

Table 4 compares the BPI score predictions obtained by the ANN, the SVM, and the MLR models for the training set and the test set. For predicting BPI score, the ANN model had relatively larger change rates of MSE values at year 1. That is, the ANN model had better BPI score prediction capability. Apparently, the ANN model also outperformed the SVM model and the MLR model in terms of predictive accuracy. Most MAPE values obtained by the ANN model were lower than 5%, which indicated the excellent accuracy of the ANN in predicting BPI score. 

The training set was also used to calculate the variable sensitivity ratios (VSR) for the ANN model.  Table 5 presents the VSR values for the outcome variables (BPI scores) in relation to the four most influential variables. In the ANN model, pretreatment BPI score was the most influential (sensitive) parameter in terms of its effects on worst pain, average pain, present pain, and aggregated pain interference (VSR 5.83, 5.51, 5.15, and 6.07, resp.). All VSR values exceeded one, indicating that the network performs better when all variables are considered. 

Table 6 compares the MAPE values obtained by ANN and SVM models. Compared to the SVM model, the ANN model consistently obtained lower MAPE values for worst pain score (4.7% versus 6.0%), average pain (4.4% versus 5.8%), present pain (4.1% versus 5.4%), and aggregated pain interference (3.6% versus 4.8%).

## 4. Discussion

This study confirmed that, compared to the SVM model and the MLR model, the ANN model is significantly more accurate in predicting pain function (*P* < 0.001). To the best of our knowledge, this study is the first to use ANNs for analyzing predictors of BPI score after dry needling. This model was tested against actual outcomes obtained by a neural network model, a support vector machine model, and a linear regression model constructed using identical inputs. We also showed that, given the same number of demographic and clinical inputs and pretreatment BPI scores, the predictive accuracy of ANN is superior to that of SVM and MLR. 

Recently, SVM and ANN models have been used for nonlinear modeling in many fields, particularly bioinformatics [6–10]. Although the efficacy of SVM models is well established in the field of machine learning, its performance in surgical outcome prediction and prognosis has not been measured. The ANNs are adaptive models that use a dynamic approach to analyzing the risk of outcomes. That is, they perform bottom-up computation by modifying their internal structures in relation to a functional objective (i.e*., *the model is generated by the data it analyzes). Despite their incapability to deal with missing data, ANNs can simultaneously process numerous variables and can consider outliers and nonlinear interactions among variables. Unlike standard statistical tests, ANNs effectively manage complexity even when samples sizes are small and when ratios between variables and records are unbalanced. In this respect, ANNs avoid the dimensionality problem and can achieve a predictive accuracy superior to those of SVM and MLR. To ensure a sufficiently robust basis for network training, the present study used a large and homogeneous dataset comprising all demographic and clinical variables shown to affect patient-reported BPI scores in previous linear regression models [9].

Throughout this one-year follow-up study, the best single predictor of BPI scores was pretreatment pain function, which is consistent with reports that pretreatment functional scores are the best predictors of posttreatment QOL [2, 9]. Therefore, effective counseling is essential for apprising patients of expected posttreatment impairments. If QOL outcomes are considered benchmarks, then pretreatment functional status, which is a major predictor of posttreatment outcome, is crucial. Patients should also be advised that their posttreatment QOL might depend not only on the success of their treatments, but also on their pretreatment functional status. 

To identify prognostic indicators after dry-needling protocol, prospective cohort follow-up studies are essential for identifying prognostic predictors. The results of the authors' analyses also showed the importance of baseline pain intensity in predicting outcomes. In agreement with previous studies, baseline pain intensity was powerful outcome predictor of musculoskeletal pain across different regional pain sites [2, 20]. More pain predicted a lower probability of recovery at followup. Having more pain and disability at baseline will leave room for a larger reduction at follow-up. Although, this does not necessarily result in a better prognosis in terms of recovery, the pain intensity may still be relatively high at followup. For example, a patient with a baseline pain score of 9 and a follow-up score of 5 improved more than a patient with a baseline score of 4 and a follow-up score of 1 [21].

Sleep deprivation does produce hyperalgesic changes in healthy subjects [22], which likely reflect alterations in supraspinal modulation of nociception such as impaired function of inhibitory modulation pathways. Sleep deprivation has a much larger effect on muscle nociception than on skin nociception [23]. Furthermore, sleep deprivation is known to produce additional effects such as increased fatigue and negative mood, which might cause a modulation of pain processing. Depression, which is strongly associated with poor mental QOL, is only moderately associated with poor physical QOL [24, 25]. However, sleep problems are apparently related to poor physical QOL [24, 25]. In the present study, sleep deprivation was significantly and positively related to BPI scores. 

Longer pain duration at baseline was indicative of poor prognosis for spinal pain, low back pain, shoulder pain, and hip pain [26]. ANN model was used to predict BPI scores, and this study results showed that pain duration was secondary rank factor for aggregated pain interference. Atroshi et al., using the SF-36 to investigate long-term sick leave among primary care patients with musculoskeletal disorders, found the long-term sick listed patients had significantly worse physical and mental health [27]. Among longer pain duration patients, physical functioning scores had not improved at one year despite a small to moderate improvement in pain scores. 

The ANN approach developed in this study extends the predictive range of the linear regression model by replacing identity functions with nonlinear activation functions. The approach is apparently superior to linear regression for describing systems. The ANNs may be trained with data acquired in various clinical contexts and can consider local expertise, racial differences, and other variables with uncertain effects on clinical outcomes. The analysis need not be limited to clinical parameters. Other potentially useful variables could be tested to improve the predictive value of the model. The proposed ANN architecture with MLP can also include more than one dependent variable and can perform a nonlinear transformation between dependent variables. Future studies may evaluate how other demographic or clinical characteristics affect the proposed architecture.

Although all research questions were satisfactorily addressed, several limitations are noted. This study collected data for myofascial pain control after dry needling under the supervision of two physicians in one medical center, each of whom had performed the highest volume of myofascial pain control after dry needling during the previous years. This sample selection procedure ensured that patient's outcome data would not be affected by physicians with limited experience. However, a notable limitation is that the first patient in the prospective patient cohort was enrolled in 2008. Therefore, depending on their inclusion date, some MPS patients had a longer followup than others did, which may have caused selection bias. Nonetheless, in most QOL subscales, the characteristics of subjects who continuously participated throughout this one-year study did not significantly differ from those of subjects who died or dropped out during the study (data not shown). 

## 5. Conclusions

Compared with the SVM model and the MLR model, the ANN model in the study was more accurate in predicting patient-reported QOL and had higher overall performance indices. The global sensitivity analysis also showed that pretreatment pain function is the most important predictor of BPI scores after dry needling. The predictors analyzed in this study could be addressed in pretreatment and posttreatment health care consultations to educate candidates for MPS patients dry needling in the expected course of recovery and expected functional outcomes. Further studies of this model may consider the effect of a more detailed database that includes complications and clinical examination findings as well as more detailed outcome data. Hopefully, the model will evolve into an effective adjunctive clinical decision making tool. 

## Figures and Tables

**Table 1:** Patient characteristics of analyzed subjects (*N* = 400).

Variables	*N* (%) or means ± SD
Age, years	48.57 ± 12.63
Pain duration, months	42.53 ± 40.21
Gender	
Female	283 (71.0%)
Male	117 (29.0%)
Marital status	
Single	96 (23.9%)
Married	304 (76.1%)
Education	
No formal education/primary school	100 (25.0%)
Junior high school	152 (38.0%)
Senior high school/college	148 (37.0%)
Drinking	
Yes	41 (10.3%)
No	359 (89.7%)
Smoking	
Yes	35 (8.8%)
No	365 (91.2%)
Sleep deprivation	
Yes	116 (29.0%)
No	284 (71%)
Nutritional deficiency	
Yes	27 (6.8%)
No	373 (93.2%)
Pretreatment pain intensity: worst, score	5.97 ± 1.72
Pretreatment pain intensity: least, score	2.18 ± 1.83
Pretreatment pain intensity: average, score	4.41 ± 1.67
Pretreatment pain intensity: present, score	4.11 ± 3.47
Pretreatment aggregated pain interference, score^#^	3.15 ± 2.03

SD: standard deviations.

^#^Aggregated pain interference was calculated as follows: [(pain interference of general activity + mood + walking ability + normal work + relationship + sleep + enjoyment of life)/7].

**Table 2:** Coefficients of significant variables for Brief Pain Inventory (BPI) scores in multiple linear regression model after dry needling.

	Worst pain		Average pain		Present pain		Aggregated pain interference*	
Variables	Coefficients	*P* value	Coefficients	*P* value	Coefficients	*P* value	Coefficients	*P* value
Age	−0.03	0.041	−0.04	0.045	−0.03	0.044	−0.05	0.029
Pain duration	0.28	<0.001	0.01	0.021	0.03	0.037	0.01	0.035
Gender (female versus male)	−0.51	0.036	−0.64	0.023	−0.69	0.031	−0.71	0.030
Marital status (single versus married)	0.54	0.039	0.67	0.014	0.68	0.014	0.59	0.038
Sleep deprivation (yes versus no)	1.53	<0.001	1.14	<0.001	0.98	0.012	1.06	<0.001
Nutritional deficiency (yes versus no)	1.82	<0.001	1.60	0.018	1.90	<0.001	1.71	0.001
Pretreatment BPI score	0.58	<0.001	0.34	<0.001	0.46	<0.001	0.28	<0.001

*Aggregated pain interference was calculated as follows: [(pain interference of general activity + mood + walking ability + normal work + relationship + sleep + enjoyment of life)/7].

**Table 3:** Three-layer networks and number of support vectors for Brief Pain Inventory (BPI) scores in artificial neural network (ANN) and support vector machine (SVM) models.

Subscales	ANN-based model*	SVM-based model^#^
Worst pain	11-5-1	143
Average pain	11-7-1	93
Present pain	11-5-1	119
Aggregated pain interference	11-4-1	127

*Values are for input layer-hidden layer-output layer.

^#^Values are numbers of support vectors.

**Table 4:** Comparison of multiple linear regression (MLR), support vector machine (SVM), and artificial neural network (ANN) models in predicting Brief Pain Inventory (BPI) scores.

Indices	Models	Training set (*A*)	Testing set (*B*)	Change rate^#^
Worst pain				
MSE	MLR	22.41	24.37	8.7%
	SVM	16.05	14.52	10.5%
	ANN	15.02	12.63	20.3%
MAPE	MLR	8.5%	8.1%	—
	SVM	5.9%	5.1%	—
	ANN	4.4%	4.5%	—

Average pain				
MSE	MLR	19.19	17.84	7.6%
	SVM	13.93	12.86	8.3%
	ANN	13.26	11.56	14.7%
MAPE	MLR	6.4%	6.2%	—
	SVM	5.5%	5.9%	—
	ANN	4.0%	4.1%	—

Present pain				
MSE	MLR	17.68	18.82	6.1%
	SVM	12.06	13.01	7.3%
	ANN	10.31	11.16	7.6%
MAPE	MLR	6.9%	6.9%	—
	SVM	5.7%	5.0%	—
	ANN	4.6%	4.4%	—

Aggregated pain interference				
MSE	MLR	14.83	14.28	3.9%
	SVM	11.06	10.18	8.6%
	ANN	8.13	8.91	8.8%
MAPE	MLR	5.6%	5.4%	—
	SVM	4.5%	4.7%	—
	ANN	3.4%	3.4%	—

MSE: mean square error, MAPE: mean absolute percentage error.

^#^Change rate = |(*B* − *A*)/(*A*)| × 100%.

**Table 5:** Global sensitivity analysis of artificial neural network (ANN) model in predicting Brief Pain Inventory (BPI) scores.

ANN model	Rank 1st	Rank 2nd	Rank 3rd	Rank 4th
	VSR	VSR	VSR	VSR
Worst pain	Pretreatment worst pain score (5.83)	Sleep deprivation (1.74)	Pain duration (1.43)	Nutritional deficiency (1.24)
Average pain	Pretreatment average pain score (5.51)	Sleep deprivation (1.66)	Pain duration (1.52)	Nutritional deficiency (1.44)
Present pain	Pretreatment present pain score (5.15)	Sleep deprivation (1.60)	Pain duration (1.20)	Nutritional deficiency (1.18)
Aggregated pain interference	Pretreatment aggregated pain interference score (6.07)	Pain duration (1.57)	Sleep deprivation (1.44)	Nutritional deficiency (1.30)

VSR: Variable sensitivity ratios.

**Table 6:** Comparison of mean absolute percentage error (MAPE) in Brief Pain Inventory (BPI) scores predicted by multiple linear regression (MLR), support vector machine (SVM) and artificial neural network (ANN) models in forty new data sets.

Models	MAPE
Worst pain	
MLR model	8.2%
SVM model	6.0%
ANN model	4.7%
Average pain	
MLR model	6.7%
SVM model	5.8%
ANN model	4.4%
Present pain	
MLR model	6.8%
SVM model	5.4%
ANN model	4.1%
Aggregated pain interference	
MLR model	5.7%
SVM model	4.8%
ANN model	3.6%

## References

[1] Olivares PR, Gusi N, Parraca JA, Adsuar JC, Del Pozo-Cruz B (2011). Tilting whole body vibration improves quality of life in women with fibromyalgia: a randomized controlled trial. *Journal of Alternative and Complementary Medicine*.

[2] Huang Y-T, Lin S-Y, Neoh C-A, Wang K-Y, Jean Y-H, Shi H-Y (2011). Dry needling for myofascial pain: prognostic factors. *Journal of Alternative and Complementary Medicine*.

[3] Kalichman L, Vulfsons S (2010). Dry needling in the management of musculoskeletal pain. *Journal of the American Board of Family Medicine*.

[4] Vulfsons S, Ratmansky M, Kalichman L (2012). Trigger point needling: techniques and outcome. *Current Pain and Headache Reports*.

[5] Hsieh Y-L, Chou L-W, Joe Y-S, Hong C-Z (2011). Spinal cord mechanism involving the remote effects of dry needling on the irritability of myofascial trigger spots in rabbit skeletal muscle. *Archives of Physical Medicine and Rehabilitation*.

[6] Shi HY, Tsai JT, Chen YM, Culbertson R, Chang HT, Hou MF (2012). Predicting two-year quality of life after breast cancer surgery using artificial neural network and linear regression models. *Breast Cancer Research and Treatment*.

[7] Zhao H, Wang W, Hou N (2011). Discovery of diagnosis pattern of coronary heart disease with Qi deficiency syndrome by the T-test-based Adaboost algorithm. *Evidence-Based Complementary and Alternative Medicine*.

[8] Tolambiya A, Thomas E, Chiovetto E, Berret B, Pozzo T (2011). An ensemble analysis of electromyographic activity during whole body pointing with the use of support vector machines. *PLoS ONE*.

[9] Shi HY, Lee HH, Tsai JT (2012). Comparisons of prediction models of quality of life after laparoscopic cholecystectomy: a longitudinal prospective study. *PLoS ONE*.

[10] Zick SM, Alrawi S, Merel G (2011). Relaxation acupressure reduces persistent cancer-related fatigue. *Evidence-Based Complementary and Alternative Medicine*.

[11] Zurada JM (1992). *Introduction to Artificial Neural Systems*.

[12] Rughani AI, Dumont TSM, Lu Z (2010). Use of an artificial neural network to predict head injury outcome: clinical article. *Journal of Neurosurgery*.

[13] Lee D, Xu H, Lin JG, Watson K, Wu RSC, Chen K-B (2011). Needle-free electroacupuncture for postoperative pain management. *Evidence-Based Complementary and Alternative Medicine*.

[14] Lin S-Y, Neoh C-A, Huang Y-T, Wang K-Y, Ng H-F, Shi H-Y (2010). Educational program for myofascial pain syndrome. *Journal of Alternative and Complementary Medicine*.

[15] Zhang SP, Yip T-P, Li Q-S (2011). Acupuncture treatment for plantar fasciitis: a randomized controlled trial with six months follow-up. *Evidence-Based Complementary and Alternative Medicine*.

[16] Simons DG, Travell JG, Simons LS (1999). Travell & Simons’s myofascial pain and dysfunction: the trigger point manual. *Upper Half of Body*.

[17] Hublin C, Kaprio J, Partinen M, Koskenvuo M (2001). Insufficient sleep—a population-based study in adults. *Sleep*.

[18] Ger L-P, Ho S-T, Sun W-Z, Wang M-S, Cleeland CS (1999). Validation of the brief pain inventory in a Taiwanese population. *Journal of Pain and Symptom Management*.

[19] Shi H-Y, Lee K-T, Lee H-H (2012). Comparison of artificial neural network and logistic regression models for predicting in-hospital mortality after primary liver cancer surgery. *PLoS ONE*.

[20] Cheung WY, Le LW, Gagliese L, Zimmermann C (2011). Age and gender differences in symptom intensity and symptom clusters among patients with metastatic cancer. *Supportive Care in Cancer*.

[21] Bot SDM, van der Waal JM, Terwee CB (2005). Predictors of outcome in neck and shoulder symptoms: a cohort study in general practice. *Spine*.

[22] Onen SH, Alloui A, Gross A, Eschallier A, Dubray C (2001). The effects of total sleep deprivation, selective sleep interruption and sleep recovery on pain tolerance thresholds in healthy subjects. *Journal of Sleep Research*.

[23] Mense S (2000). Neurobiological concepts of fibromyalgia—the possible role of descending spinal tracts. *Scandinavian Journal of Rheumatology, Supplement*.

[24] Castro-Sánchez AM, Matarán-Pearrocha GA, Granero-Molina J, Aguilera-Manrique G, Quesada-Rubio JM, Moreno-Lorenzo C (2011). Benefits of massage-myofascial release therapy on pain, anxiety, quality of sleep, depression, and quality of life in patients with fibromyalgia. *Evidence-based Complementary and Alternative Medicine*.

[25] Tsauo J-Y, Lin K-Y, Hu Y-T, Chang K-J, Lin H-F (2011). Effects of yoga on psychological health, quality of life, and physical health of patients with cancer: a meta-analysis. *Evidence-based Complementary and Alternative Medicine*.

[26] Mallen CD, Peat G, Thomas E, Dunn KM, Croft PR (2007). Prognostic factors for musculoskeletal pain in primary care: a systematic review. *British Journal of General Practice*.

[27] Atroshi I, Andersson IH, Gummesson C, Leden I, Odenbring S, Ornstein E (2002). Primary care patients with musculoskeletal pain: value of health-status and sense-of-coherence measures in predicting long-term work disability. *Scandinavian Journal of Rheumatology*.

